# Genes encoding critical transcriptional activators for murine neural tube development and human spina bifida: a case-control study

**DOI:** 10.1186/1471-2350-11-141

**Published:** 2010-10-08

**Authors:** Wei Lu, Adrian R Guzman, Wei Yang, Claudia J Chapa, Gary M Shaw, Robert M Greene, M Michele Pisano, Edward J Lammer, Richard H Finnell, Huiping Zhu

**Affiliations:** 1 Institute of Biosciences and Technology, Texas A&M University System Health Science Center, Houston, TX, USA; 2 Stanford University, Stanford, CA, USA; 3 University of Louisville Birth Defects Center, Louisville, KY, USA; 4 Children's Hospital Oakland Research Institute, Oakland, CA, USA; 5 Dell Pediatric Research Institute, UT Austin, Austin, TX, USA

## Abstract

**Background:**

Spina bifida is a malformation of the neural tube and is the most common of neural tube defects (NTDs). The etiology of spina bifida is largely unknown, although it is thought to be multi-factorial, involving multiple interacting genes and environmental factors. Mutations in transcriptional co-activator genes-*Cited2*, *p300*, *Cbp*, *Tfap2α*, *Carm1 *and *Cart1 *result in NTDs in murine models, thus prompt us to investigate whether homologues of these genes are associated with NTDs in humans.

**Methods:**

Data and biological samples from 297 spina bifida cases and 300 controls were derived from a population-based case-control study conducted in California. 37 SNPs within *CITED2*, *EP300*, *CREBBP*, *TFAP2A*, *CARM1 *and *ALX1 *were genotyped using an ABI SNPlex assay. Odds ratios and 95% confidence intervals were calculated for alleles, genotypes and haplotypes to evaluate the risk for spina bifida.

**Results:**

Several SNPs showed increased or decreased risk, including *CITED2 *rs1131431 (OR = 5.32, 1.04~27.30), *EP300 *rs4820428 (OR = 1.30, 1.01~1.67), *EP300 *rs4820429 (OR = 0.50, 0.26~0.50, in whites, OR = 0.7, 0.49~0.99 in all subjects), *EP300 *rs17002284 (OR = 0.43, 0.22~0.84), *TFAP2A *rs3798691 (OR = 1.78, 1.13~2.87 in Hispanics), *CREBBP *rs129986 (OR = 0.27, 0.11~0.69), *CARM1 *rs17616105 (OR = 0.41, 0.22~0.72 in whites). In addition, one haplotype block in *EP300 *and one in *TFAP2A *appeared to be associated with increased risk.

**Conclusions:**

Modest associations were observed in *CITED2*, *EP300*, *CREBBP*, *TFAP2A *and *CARM1 *but not *ALX1*. However, these modest associations were not statistically significant after correction for multiple comparisons. Searching for potential functional variants and rare causal mutations is warranted in these genes.

## Background

Studies using genetically modified mouse models have contributed a great deal to our understanding of molecular mechanisms that govern critical embryonic development processes, such as neural tube closure. Among the over 200 existing mouse models for neural tube defects (NTDs), several harbor mutations in genes encoding transcriptional activators or co-activators, such as *Cited2*, *Crebbp*, *p300*, *Tfap2α, Carm1 *and *Cart1 *[1-9].

CREB binding protein (CREBBP; CBP) was originally identified as a protein that binds to the phosphorylated form of the CREB transcription factor and increases the expression of genes containing CRE elements. It is closely related to the adenovirus E1A-associated protein p300. CBP and p300 are functionally redundant histone deacetylases essential for proliferation and embryonic development [5,8,10]. Both *Crebbp *and *p300 *genes are expressed at high level in the neural folds at embryonic day E8.5 in mice [3]. Homozygous deletions of either the *Crebbp *or *p300 *genes in mice results in lethality on E9~11, with subsequent defects in neurulation, cell proliferation and cardiac development [5,8].

The methylation of the Crebbp/p300 complex is catalyzed by co-activator-associated arginine methyltransferase (CARM1). This process disables the recruitment of cAMP response element binding protein (CREB). Thus, CARM1 functions as a co-repressor in the cAMP signaling pathway, via its methyltransferase activity [11]. In addition to its enzymatic function, CARM1 is also required for NF-kappaB-dependent gene expression as a transcriptional cofactor [12]. *Carm1 *gene expression was observed in the fore- and hindbrain, neural folds, somites and posterior lateral plates of E8.25 mouse embryos, and later on in development (E8.75) expression became prominent in the neural tube and somites [13].

CITED2 is a nuclear co-activator that binds to CBP/p300. Disruption of the *Cited2 *gene in mice is embryonic lethal, with the mice dying secondary to significant defects in the development of the neural tube and the heart [2,6,14,15]. The interaction of CBP and p300 with *Cited2 *is required for activation of important transcriptional factors such as AP-2α [1,16]. Mice carrying homozygous disruptions of the gene coding for the protein AP-2 α (*Tfap2 *α) die at term as a result of severe developmental abnormalities. The earliest defect observed was a complete failure of neural tube closure in the cranial region, and it has been suggested in a chimera model study that there is an independent requirement for AP-2 α in the formation of the neural tube [17-19].

CART1 is a paired-class homeobox-containing transcription factor regulated by CBP/p300 acetylation of a highly conserved lysine residue that increases the interaction between CBP/p300 and CART1, ultimately enhancing its transcriptional activation [20]. The *Cart1 *gene is expressed in chondrocytes [21,22], and appears to be required for embryonic forebrain mesenchymal survival. The absence of CART1 disrupts cranial neural tube morphogenesis by blocking the initiation of closure in the midbrain region. Homozygous *Cart1 *mutant mice are born alive with neural tube closure defects including acrania and meroanencephaly, but die soon after birth [9]. The *Cart1 *knockout mouse is among several NTD mouse models that can be rescued by maternal supplementation with folic acid [23]. The gene encoding human CART1 is *ALX1 *(ALX homobox 1). In summary, CBP, p300, CARM1, TFAP2α, CITED2 and CART1 are transcriptional activator/co-activators that contributes to the ontogeny of the neural tube in mammalian systems. Whether gene homologues are associated with human NTDs remains unknown. Three single nucleotide polymorphisms (SNP) in the un-translated region of *CITED2 *gene were studied in a group of patients with spina bifida and controls, modest associations were observed but results were imprecise owing to small sample size [14]. Mutations in the human *CBP *gene have been identified as being responsible for the Rubinstein-Taybi syndrome (RTS), which has a defined craniofacial phenotype [4]. The purpose of this study is to evaluate the potential association between variations among human *CITED2*, *CREBBP*, *EP300*, *TFAP2A*, *CARM1 *and *ALX1 *genes and the risk for spina bifida.

## Methods

### Study Population

Data and biological samples were obtained from a case-control study conducted by the California Birth Defects Monitoring Program (CBDMP). The CBDMP is an active, population-based surveillance system for collecting information on infants and fetuses with structural congenital malformations, which has been described elsewhere [24]. Included were 297 singleton infants with spina bifida (cases) and 300 non-malformed infants (controls). Both cases and controls were randomly selected from the samples with bloodspots available. Cases were selected from California Birth Defect Registry 1990-1999; non-malformed controls were selected from Vital Statistics matching the birth years with the selected cases. Case and control infants were linked to their newborn screening bloodspots. All samples were obtained with approval from the State of California Health and Welfare Agency Committee for the Protection of Human Subjects.

### Candidate genes and SNPs

The 6 candidate genes and SNPs genotyped are listed in Table 1. Tagging SNPs were selected for each candidate gene using the SNPbrowser™ Software (ABI, Foster City, CA) based on data from the four HapMap populations (CEU, YRI, JPT and CHB) [25]. All validated tagging SNPs with pairwise r^2 ^> = 0.8 with a minor allele frequency (MAF) no less than 5% were included for genotyping. Also included for study were all non-synonymous coding SNPs in these genes. Based on these criteria, 37 SNPs were genotyped.

**Table 1:** Characteristics of all genotyped SNPs

Gene (Alias)	Marker	Chromosome	Chromosome position	RefSNP alleles	Call Rate (%)	MAF* (All subjects)	HWE P (controls)	MAF (White)	HWE** P (White controls)	MAF (Hispanic)	HWE P (Hispanic controls)
*ALX1 (CART1)*	rs11608773	12	84229006	A/G	100.0	0.229	0.435	0.274	0.478	0.217	0.391
	
	rs4761130	12	84226396	C/T	99.8	0.421	0.286	0.358	0.154	0.401	0.465
	
	rs7295242	12	84222948	A/G	100.0	0.161	0.781	0.074	0.557	0.159	0.358

*CARM1*	rs11667234	19	10880581	A/C	99.8	0.021	0.010	0.039	0.071	0.016	0.934
	
	rs11670365	19	10865833	C/T	100.0	0.056	0.918	0.084	0.754	0.045	0.702
	
	rs1529711	19	10884434	A/G	99.7	0.097	0.492	0.161	0.932	0.081	0.519
	
	rs1541596	19	10848013	A/G	99.5	0.475	0.326	0.497	0.969	0.408	0.957
	
	rs17616105	19	10883159	C/T	100.0	0.189	0.732	0.235	0.365	0.152	0.072
	
	rs7254708	19	10863579	A/G	100.0	0.071	0.043	0.123	0.116	0.059	0.441

*CITED2*	rs1131431	6	139735527	A/G	100.0	0.153	0.273	0.200	0.624	0.121	0.948
	
	**rs7775752**	6	139737486	**A/G**	**45.1**	**0.007**	**0.957**	**0.009**	**0.913**	**0.006**	**1.000**

*CREBBP (CBP)*	rs11076783	16	3722706	C/T	100.0	0.154	0.638	0.116	0.142	0.139	0.802
	
	**rs11076785**	16	3730187	**G/T**	**46.6**	**0.457**	**0.638**	**0.401**	**0.185**	**0.457**	**0.463**
	
	rs11076786	16	3751597	C/T	100.0	**0.075**	**0.008**	0.077	0.441	0.046	0.637
	
	rs129986	16	3744242	C/T	100.0	0.023	0.530	0.032	0.590	0.020	0.735
	
	rs130003	16	3768173	A/G	99.8	0.015	0.792	0.032	0.711	0.010	0.967
	
	rs130008	16	3721953	C/G	99.8	0.011	0.838	0.032	0.752	0.001	1.000
	
	rs17136507	16	3740546	C/T	100.0	0.178	0.536	0.110	0.229	0.227	0.449
	
	rs886528	16	3751557	C/T	99.7	0.383	0.590	0.390	0.986	0.368	0.839
	
	rs9392	16	3715170	A/G	99.8	0.189	0.566	0.223	0.625	0.138	0.700

*EP300 (P300)*	rs17002284	22	39826792	A/G	100.0	0.265	0.220	0.313	0.745	0.217	0.522
	
	rs17433014	22	39829768	A/G	99.8	0.018	0.769	0.029	0.793	0.019	0.834
	
	rs2230111	22	39851949	A/G	99.8	0.002	0.977	0.006	0.959	1.000	1.000
	
	rs4820428	22	39867535	A/G	100.0	**0.446**	**0.006**	0.329	0.822	0.419	0.667
	
	rs4820429	22	39868057	C/G	100.0	0.159	0.382	0.258	0.040	0.124	0.471
	
	rs4822006	22	39849308	C/T	100.0	0.247	0.844	0.355	0.587	0.185	0.740
	
	rs5758246	22	39871792	A/G	100.0	0.030	0.635	1.000	1.000	0.012	0.934
	
	rs9611502	22	39861182	C/T	100.0	0.014	0.838	0.019	0.918	0.013	0.867

*TFAP2A (AP2)*	rs10456013	6	10506548	G/T	100.0	0.032	0.592	0.048	0.670	0.026	0.735
	
	rs1621700	6	10528084	A/G	99.7	0.288	0.671	0.252	0.081	0.357	0.773
	
	rs303050	6	10511169	C/T	100.0	0.257	0.061	0.165	0.259	0.262	0.267
	
	rs303055	6	10527462	C/T	100.0	0.353	0.150	0.435	0.569	0.335	0.025
	
	rs303056	6	10528467	C/G	100.0	0.159	0.229	0.094	0.477	0.126	1.000
	
	rs3798691	6	10519381	C/G	100.0	0.112	0.021	0.042	0.011	0.134	0.111
	
	rs3798694	6	10516743	A/G	100.0	0.062	0.122	0.087	0.225	0.059	0.293
	
	rs533558	6	10503558	A/G	100.0	0.441	0.848	0.361	0.955	0.481	0.858
	
	rs537112	6	10503192	A/G	100.0	0.180	0.571	0.103	0.754	0.195	0.255

*MAF: minor allele frequency; **HWE: Hardy-Weinberg Disequilibrium

### Genotyping Approach

DNA was extracted from blood spots using the Gentra Puregene DNA Extraction Kit (Qiagen, Valencia, CA) following the standard protocol. Prior to genotyping, genomic DNA was amplified using a commercially available multiple displacement amplification (MDA) kit, GenomiPhi DNA Amplification kit (GE Healthcare, Piscataway, NJ). The whole genome amplification (WGA) product was then quantified using RNase P assay (AppliedBiosystems, Foster City, CA). 200 ng of WGA product was used for the SNPlex assay containing the 37 SNPs. All genotyping assays were performed blinded to subjects' case or control status.

The SNPs selected for analyses were submitted to Applied Biosystems to create a unique SNPlex assay. The SNPlex™ genotyping system (Applied Biosystems, Foster City, CA) is based on a universal multiplexed oligonucleotide ligation assay (OLA)/PCR and drag chute mobility modifier technology. The resulting genotype data were analyzed using GeneMapper™ v4.0 (Applied Biosystems, Foster City, CA).

### Analytic procedures

Analyses were performed for the overall study group as well as for specific ethnic/race groups. Hispanic infants were further subdivided into those whose mothers were born in the United States, or native-born (Hisp-NB) and those whose mothers were not, or foreign-born (Hisp-FB), as it has been shown that these two groups are different with respect to ethnic composition and social economic status [26,27]. Departure from Hardy-Weinberg Equilibrium (HWE) was assessed by Chi-square analysis in each subgroup using data from controls only. Odds ratios (OR) for SNP genotypes were calculated using logistic regression analyses. Since the mode of inheritance is unknown, potential associations with spina bifida risk were evaluated assuming dominant, recessive and additive models for each SNP. Chi-square tests were used to assess the significance of dominant and recessive models. Cochran-Armitage trend tests were used to assess the significance of additive models. If numbers were less than 5, Fisher's exact test and Exact Armitage trend test were applied. Analyses were performed using SVS v7.1.1 (GoldenHelix, Bozeman, MT) software. The false discovery rate (FDR) [28] was used to account for multiple testing (data not shown). Haplotype blocks were constructed using HaploView 4.2 http://www.broadinstitute.org/haploview/haploview in each race/ethnicity group with pair-wise D'≥0.85. Haplotype frequencies were estimated based on the Maximum Expectation (EM) algorithm. Association of each haplotype was evaluated by calculating ORs and 95% confidence intervals against all other haplotypes. Estimated risks were considered statistically precise if 95% confidential interval of OR does not include 1.00.

## Results and Discussion

Demographic characteristics of the cases and controls are listed in Table 2. Cases and controls were similar with respect to maternal age, parity, and plurality but expectedly differed with respect to race/ethnicity and maternal educational attainment. That is, the data revealed greater frequencies of Hispanics (primarily foreign born) and women with low educational level (less than high school) in the spina bifida case group.

**Table 2:** Demographic distribution of malformed cases and non-malformed controls, California 1990-1999

Variable	Spina Bifida	
	
	Cases **n = 297 (%**^**1**^**)**	Controls **n = 300 (%**^**1**^**)**
Maternal Race/Ethnicity		
White, non-Hispanic	59 (19.9)	96 (32.0)
Hispanic, Native Born	33 (11.1)	33 (11.0)
Hispanic, Foreign Born	166 (55.9)	115 (38.3)
Black	17 (5.7)	20 (6.7)
Asian	9 (3.0)	25 (8.3)
Other	9 (3.0)	11 (3.7)
Maternal age at delivery (years)		
13-24	128 (43.1)	123 (41.0)
25-29	80 (26.9)	71 (23.7)
30-34	47 (15.8)	64 (21.3)
35-55	39 (13.1)	42 (14.0)
Maternal education		
Less than high school	158 (53.2)	110 (36.7)
High School	65 (21.9)	81 (27.0)
Greater than High school	71 (23.9)	109 (36.3)
Parity		
0	120 (40.4)	114 (38.0)
1	88 (29.6)	101 (33.7)
≥ 2	86 (29.0)	84 (28.0)
Plurality		
Singleton	287 (96.6)	293 (97.7)
Multiple births	8 (2.7)	7 (2.3)
Infant Sex		
Male	146 (49.2)	149 (49.7)
Female	151 (50.8)	151 (50.3)

^1^Percentages may not equal 100 owing to missing data or rounding.

Genotyping of 37 SNPs was performed using a custom-made Applied Biosystems SNPlex panel. Due to low call rates, two SNPs, rs7775752 (45.1%) and rs11076785 (46.6%), were excluded from analysis. Departure from HWE in the controls (*p *< 0.01) was observed in rs11076786 and rs4820428 when analyzing the whole dataset. This deviation was eliminated when stratified by race/ethnicity (Table 1). Several SNPs and haplotypes containing these SNPs showed modest associations with spina bifida risk (p ≤ 0.05, Table 3 and 4). These associations are summarized below for each gene. The results, however, are largely indicative of no or very limited associations, particularly after correction for multiple comparisons.

**Table 3:** Allele associations (P < 0.05) among subgroups

SNP	Gene	Ethnicity	refSNP alleles	Minor Allele	Ratio Counts (Case, Control)	P value	Odds Ratio	95% CI
rs129986	*CREBBP (CBP)*	white	C/T	T	118:0, 182:10	0.008*	n/a	n/a

rs17616105	*CARM1*	white	C/T	C	101:17, 136:56	0.003	2.45	1.34-4.46

rs303056	*TFAP2A (AP2)*	white	C/G	C	16:102, 13:179	0.046	2.16	1.00-2.15

rs3798691	*TFAP2A (AP2)*	Hispanic	C/G	C	65:333, 29:267	0.013	1.80	1.13-2.87
		
		Hispanic-FB	C/G	C	52:280, 22:208	0.036	1.76	1.03-2.98

rs533558	*TFAP2A (AP2)*	Hispanic-NB	A/G	G	36:30, 24:42	0.036	2.10	1.05-4.22

rs4820428	*EP300 (P300)*	All subjects	A/G	G	276:240, 229:259	0.038	1.30	1.01-1.67

**Table 4:** Genotype associations (P < 0.05) among subgroups under different modes of inheritance

Marker	Gene	Ethnicity	*P *value	Mode of Inheritance	Odds Ratio [(ratio count (case, control))	95% CI
rs17002284	*EP300*	All subjects	0.012	Recessive	**0.43 (13, 29; 284, 271)**	**0.22 - 0.84**

rs4820428	*EP300*	All subjects	0.011	Additive [(Dd) vs (dd)]	**1.46 (132, 122; 87, 117)**	**1.00 - 2.11**
			
				[(DD) vs (Dd)]	1.18 (78, 61; 132, 122)	0.78 - 1.79

		All subjects	0.012	Dominant	**1.54 (210, 183; 87, 117)**	**1.10 - 2.17**

rs4820429	*EP300*	All subjects	0.046	Dominant	**0.70 (75, 98; 222, 202)**	**0.49 - 0.99**

		White	0.041	Dominant	**0.50 (22, 52; 37, 44)**	**0.26 - 0.50**

rs1621700	*TFAP2A*	All subjects	0.041	Additive [(Dd) vs (dd)]	1.30 (131, 118; 138, 161)	0.92 - 1.81
			
				[(DD) vs (Dd)]	1.33 (28, 19; 131, 118)	0.70 - 2.50

rs3798691	*TFAP2A*	Hispanic	0.015	Additive [(Dd) vs (dd)]	**1.78 (48, 23; 143, 122)**	**1.04 - 3.19**
			
				[(DD) vs (Dd)]	1.40 (9, 3; 47, 22)	0.35 - 5.70

		Hispanic	0.022	Dominant	**1.84 (56, 26; 143, 122)**	**1.13 - 3.25**

		Hispanic-FB	0.049	Dominant	**1.82 (44, 19; 122, 92)**	**1.00 - 3.40**

rs533558	*TFAP2A*	Hispanic-NB	0.038	Additive [(Dd) vs (dd)]	3.00 (18, 14; 6, 14)	0.92 - 9.80
			
				[(DD) vs (Dd)]	1.40 (9, 5; 18, 14)	0.38 - 5.12

		Hispanic-NB	0.032	Dominant	**3.32 (27, 19; 6, 14)**	**1.08 - 10.18**

rs17616105	*CARM1*	Hispanic	0.0	Recessive	n/a (6, 0; 193, 148)	n/a

		White	0.004	Additive [(Dd) vs (dd)]	0.48 (15, 36; 43,50)	0.23 - 1.00

				[(DD) vs (Dd)]	0.24 (1,10; 15,36)	0.03 - 2.04

		White	0.010	Dominant	**0.40 (16, 46; 43, 50)**	**0.40 - 0.20**

rs1131431	*CITED2*	White	0.027	Recessive	**5.32 (6, 2; 53, 94)**	**1.04 - 27.30**

rs129986	*CREBBP*	All subjects	0.005	Additive [(Dd) vs (dd)]	**0.27 (6, 21; 291, 279)**	**0.11 - 0.69**
			
				[(DD) vs (Dd)]	n/a (0, 0; 6, 21)	n/a

		All subjects	0.003	Dominant	**0.27 (6, 21; 291, 279)**	**0.11 - 0.69**

		White	0.014	Additive [(Dd) vs (dd)]	n/a (0, 10; 59, 86)	n/a
			
				[(DD) vs (Dd)]	n/a (0, 0; 0, 10)	n/a

		White	0.010	Dominant	n/a (0, 10; 59, 86)	n/a

### CITED2

Only one of the two SNPs genotyped was fully analyzed. SNP rs1131431 showed a genotypic association (OR = 5.32, 95% CI: 1.04~27.30) based on a recessive mode of inheritance, specifically in the White population. It is noteworthy that the confidence interval had a wide range, suggesting that the estimate may be imprecise due to the small number of White cases and controls with the variant. This SNP, however, was previously associated with a modest increase in risk for spina bifida and congenital heart defects [14].

### EP300

Among the 8 SNPs analyzed, the G allele of SNP rs4820428 was over-represented in spina bifida cases (OR = 1.30, 95% CI: 1.01~1.67) in all subjects. Analyses showed that rs4820428 was associated with spina bifida risk under a dominant model (OR = 1.54 95%CI: 1.10~2.17). The minor allele of rs4820429 (C) showed a reduced risk in the Whites (OR = 0.50, 95% CI: 0.26~0.50) and in the whole dataset (OR = 0.70, 95% CI: 0.49~0.99) under a dominant model. Minor allele of rs17002284 (G) also showed a reduced risk (OR = 0.43, 95%CI: 0.22~0.84), but under a recessive model. These three SNPs were in linkage disequilibrium. Subsequent analysis showed that haplotype ACGG (rs17002284-rs4822006-rs4820428-rs4820429) accounted for 50.3% of all haplotypes in the Whites and was over-represented in the white cases (OR = 1.30, 95%CI: 1.01~1.67). It is noteworthy that this high risk haplotype contains the high risk alleles for rs17002284 (A), rs4820428 (G) as well as rs4820429 (G) (Table 5).

**Table 5:** Haplotype associations (only significant associations are shown)

Haplotype Block	Frequency	P Value	Odds Ratio (case, control)	95% CI
*TFAP2A*-rs537112, rs533558, rs303050, rs3798694, and rs3798691 in Hispanic				

**AGCGC**	**0.133**	**0.0205**	**1.73 (63.0 : 335.0, 29.0 : 267.0)**	**1.08-2.77**
GATGG	0.516	0.2019	0.82 (197.0 : 201.0, 161.0 : 135.0)	0.61-1.11
GGTGG	0.201	0.8135	0.96 (78.9 : 319.1, 60.8 : 235.2)	0.66-1.39
GGCAG	0.058	0.4974	1.26 (25.0 : 373.0, 15.0 : 281.0)	0.65-2.43
AGCGG	0.041	0.3207	0.69 (13.9 : 384.1, 14.8 : 281.2)	0.33-1.45
GGCGG	0.028	0.6568	0.81 (10.1 : 387.9, 9.2 : 286.8)	0.33-2.00
AGTGG	0.021	0.9639	0.97 (8.1 : 389.9, 6.2 : 289.8)	0.34-2.79

*EP300*- rs17002284, rs4822006, rs4820428 and rs4820429 in non-Hispanic white				

**ACGG**	**0.503**	**0.0377**	**1.30 (276.0 : 240.0, 229.0 : 259.0)**	**1.01-1.67**
GCAG	0.245	0.1665	0.82 (117.0 : 399.0, 129.0 : 359.0)	0.61-1.09
ATAC	0.163	0.054	0.72 (73.0 : 443.0, 91.0 : 397.0)	0.51-1.00
ATAG	0.072	0.8074	1.06 (38.0 : 478.0, 34.0 : 454.0)	0.66-1.72
ACAG	0.015	0.2348	1.91 (10.0 : 506.0, 5.0 : 483.0)	0.65-5.63

### TFAP2

Among the 9 SNPs analyzed, rs303056 (C), rs3798691 (C), rs533558 (G) showed allelic association with spina bifida risk in Hispanics. Genotypic association analysis showed that rs3798691 was associated with spina bifida risk under a dominant model in Hispanics (OR = 1.78, 95% CI: 1.13~2.87). SNP rs533558 also showed an association under a dominant model, but only in native-born Hispanics (OR = 3.32, 95%CI: 1.08~10.18). Subsequent haplotype analysis predicted two haplotype blocks in Hispanics. Haplotype AGCGC of block1 (rs537112- rs533558-rs303050-rs3798694-rs3798691) had an average frequency of 0.133 and was associated with an increased risk, OR = 1.73 (1.08-2.77) compared to all other haplotypes. Since no high risk SNP has been discovered, these high risk blocks will be thoroughly sequenced to search for other variants (Table 5).

### CREBBP

Among the 8 SNPs analyzed, the heterozygous status of SNP rs129986 (TC) was associated with a reduced risk in all subjects (OR = 0.27, 95% CI: 0.11~0.69). The homozygous status of the minor allele, CC, was not observed. The TC genotype was observed in only 6 cases and they were all Hispanics.

### CARM1

6 SNPs were genotyped. In Whites, the minor allele (C) of rs17616105 showed a reduced risk with OR = 0.41 (95%CI: 0.22~0.75). Genotype analysis showed that this effect followed a dominant inheritance model (OR_CC+CT _= 0.40, 95% CI: 0.20~0.40), and was not observed in the Hispanics.

### ALX1

3 SNPs were genotyped. No association was observed between any SNP and spina bifida risk. It is known that the *Cart1 *knockout mouse responses to maternal supplementation with folic acid [23]. The majority of the DNA samples for this study were collected after folic acid fortification is implied in the USA, which might interact with the genotyping effect; however, our study lacks the statistic power to analyze the gene-environment interaction.

To our knowledge, this is the first study evaluating human *CREBBP*, *EP300*, *CARM1*, *TFAP2A*, and *ALX1 *genes as possible risk factors for spina bifida. Association between *CITED2 *and spina bifida has been studied before, and results from our current study are consistent with the previous findings [14]. Modest associations were observed between spina bifida risk and *TFAP2A, EP300, CITED2, CREBBP1 *and *CARM1*. No association was observed in *ALX1 *gene. However, given the number of comparisons made, relatively few associations emerged. Although NTDs are multifactorial diseases, it cannot be excluded that the studied genes may contain rare mutations that cause some of the NTDs. With the most advanced next-generation sequencing technique being available, we may be able to discover such rare variants in our study population.

The strengths of this study include its population-based ascertainment of cases and controls and its evaluation of race/ethnicity as potential modifiers of risk in the presence of variant genotypes and haplotypes. A major limitation of our study was the small sample size, which reduced the statistical power of analyses. Replication of some of the modest findings in a larger sample set may be warranted.

## Conclusions

We have observed modest associations with risk of spina bifida in *CITED2*, *EP300*, *CREBBP*, *TFAP2A *and *CARM1 *genes but not *ALX1 *gene. These modest associations were not statistically significant after correction for multiple comparisons. Future effort will focus on searching for potential functional variants and rare causal mutations in these potential candidate genes.

## Abbreviations

**CREB**: cAMP response element binding protein; **CRE**: cyclic AMP-responsive; ***CITED2***: Cbp/p300-interacting transactivator, with Glu/Asp-rich carboxy-terminal domain, 2; ***EP300***: E1A binding protein p300 (Alias: p300); ***CREBBP***: CREB binding protein (Alias: CBP); ***TFAP2A***: transcription factor AP-2 alpha (activating enhancer binding protein 2 alpha) (Alias: AP-2); ***CARM1***: coactivator-associated arginine methyltransferase 1; ***ALX1***: ALX homeobox 1 (Alias: CART1).

## Competing interests

The authors declare that they have no competing interests.

## Authors' contributions

All authors have read and approved the final manuscript. WL performed and supervised most of the experiments, and also performed part of the statistical analysis. ARG and CJC performed most of the experiments and helped with manuscript writing. WY performed part of the statistical analysis and helped with the manuscript writing. GMS contributed to the study design and authorized the use of CBDMP samples. RMG and MMP contributed to candidate gene selection and study. RHF contributed to the study design and laboratory instruction of the molecular biology experiments. EJL reviewed and confirmed the clinical diagnosis of study subjects. HZ contributed to the overall study design, quality assurance of genotyping experiments, statistical analysis and most of the manuscript writing.

## Pre-publication history

The pre-publication history for this paper can be accessed here:

http://www.biomedcentral.com/1471-2350/11/141/prepub
